# Intra-host growth kinetics of dengue virus in the mosquito *Aedes aegypti*

**DOI:** 10.1371/journal.ppat.1008218

**Published:** 2019-12-02

**Authors:** Mario Novelo, Matthew D. Hall, Damie Pak, Paul R. Young, Edward C. Holmes, Elizabeth A. McGraw

**Affiliations:** 1 School of Biological Sciences, Monash University, Melbourne, Victoria, Australia; 2 Center for Infectious Disease Dynamics, Department of Entomology, The Huck Institutes of the Life Sciences, The Pennsylvania State University, University Park, Pennsylvania, United States of America; 3 Center for Infectious Disease Dynamics, Department of Biology, The Huck Institutes of the Life Sciences, The Pennsylvania State University, University Park, Pennsylvania, United States of America; 4 Australian Infectious Diseases Research Centre, School of Chemistry and Molecular Biosciences, The University of Queensland, St Lucia, Queensland, Australia; 5 Marie Bashir Institute for Infectious Diseases and Biosecurity, Charles Perkins Centre, School of Life and Environmental Sciences and Sydney Medical School, The University of Sydney, New South Wales, Australia; University of Michigan, UNITED STATES

## Abstract

Dengue virus (DENV) transmission by mosquitoes is a time-dependent process that begins with the consumption of an infectious blood-meal. DENV infection then proceeds stepwise through the mosquito from the midgut to the carcass, and ultimately to the salivary glands, where it is secreted into saliva and then transmitted anew on a subsequent bite. We examined viral kinetics in tissues of the *Aedes aegypti* mosquito over a finely graded time course, and as per previous studies, found that initial viral dose and serotype strain diversity control infectivity. We also found that a threshold level of virus is required to establish body-wide infections and that replication kinetics in the early and intermediate tissues do not predict those of the salivary glands. Our findings have implications for mosquito GMO design, modeling the contribution of transmission to vector competence and the role of mosquito kinetics in the overall DENV epidemiological landscape.

## Author summary

DENV infection in the mosquito is a complex and dynamic process. Following ingestion of an infected blood meal, DENV enters the mosquito midgut epithelial cells, where it replicates. Subsequently, the virus disseminates and infects other tissues, including hemocytes, fat body and reproductive organs, ultimately reaching the salivary glands. The kinetics of infection are influenced by genetic variation in the virus. Comparisons between strains within single serotypes, have revealed variation in infection rates in mosquitoes. To explore the role of infectious dose, serotype and tissue in viral infection kinetics we sampled DENV loads in populations of infected mosquitoes over numerous, sequential time-points. We reveal that the kinetics of DENV infection in the midgut, carcass and salivary glands of the mosquito *Aedes aegypti* are strikingly different among the strains selected for this study, and that these differences are also driven by the initial infectious dose.

## Introduction

Dengue is the most prevalent arboviral disease globally [1], with estimates of more than 390 million infections per year [2] and over half of the world’s population at risk [3]. Although mortality is generally low, the morbidity caused by dengue disease is associated with a substantial socioeconomic burden [4]. Dengue virus (DENV), transmitted to humans through the bite of the *Aedes aegypti* mosquito [5], is spreading in large part due to the expanding geographic range of the vector [6]. Previously confined to Africa, *Aedes aegypti* is now present in tropical and temperate regions worldwide, and its spread is assisted by climate change, globalization and ineffective vector control programs [7].

DENV infection in the mosquito is a complex and dynamic process [8]. The virus must circumvent multiple tissue barriers, including the midgut and salivary glands, and infect a range of intermediate tissues in a stepwise fashion [9]. Following ingestion, DENV enters the midgut epithelial cells, where it replicates. Subsequently, the virus disseminates and infects secondary tissues, including hemocytes, fat body and reproductive tissue, ultimately reaching the salivary glands [10]. The midgut is thought to represent the primary barrier to the process of infection [9], capable of preventing many mosquitoes from reaching the stage of disseminated infections [11]. The rate of this progression dictates the extrinsic incubation period (EIP), or the delay before a mosquito can infect another human on a subsequent bite [12]. The EIP plays an important role in shaping transmission rates [13], with longer time windows reducing the number of opportunities for pathogen transmission over a mosquito’s lifetime.

The kinetics of infection are equally influenced by genetic variation in the virus. Dengue fever is caused by four different serotypes of virus (DENV 1–4) [14] that are 65–70% similar at the DNA sequence level across their ~11-kb genomes [15], while also exhibiting a high average within-serotype diversity of ~3% at the amino acid level [16]. Comparisons between strains within single serotypes, have revealed variation in both infection rates and EIP in mosquitoes [17, 18], most likely due to differences in viral replication rates [11]. In humans, genetic variation within and between serotypes also determines relative viral fitness (replication rate), epidemic potential and virulence [19, 20], with particular strains linked to more severe clinical manifestations. The intra-host/vector diversity of DENV can also play a role in transmission, such as variants with a replicative advantage can spread more rapidly overall, eventually displacing those with lower fitness [21]. For example, an uncharacteristically large outbreak of dengue in Cairns, Australia in 2008/2009 was attributed to the very short EIP of the DENV-3 strain in the mosquito [22].

Surprisingly, little is known about DENV kinetics in the mosquito and how the virus interacts with individual tissues during infection. Mosquitoes have evolved both systemic and tissue specific antiviral mechanisms to limit viral replication [23]; thus, viral kinetics may differ between tissues. Given that the virus moves in a stepwise fashion, selective pressures in an initial tissue might therefore have cascading effects on the viral kinetics in downstream tissues [24]. Additionally, different tissue types may offer a diversity of cell types and cellular niches that may vary in their capacity to support DENV replication. For example, the midgut epithelium is a dynamic niche, with cells being shed frequently [25], whereas other cell types in intermediate tissues may be more stable sites of virus production.

A better understanding of intra vector kinetics will assist with developing optimal intervention points for genetic modification, improve our model of vectorial capacity [13] by adding components of dose and serotype, etc. [26] and, expand our understanding of DENV epidemiology, and vector-virus interactions such as peaks in viral load and latency periods in the host. Herein we compared midgut, carcass and salivary gland loads for 4 strains of DENV representing each of the 4 serotypes, fed at either high or low infectious doses, daily over a period of 3 weeks. In so doing, we were able to assess how the factors of dose and serotype strain diversity define susceptibility, EIP and transmissibility but also how tissue specific differences and the inter relationships between tissue kinetics drive transmissibility.

## Results

We orally challenged inbred wild-type *Ae*. *aegypti* mosquitoes with two infectious doses (10^8^ and 10^5^ DENV copies/ml), representative of plasma viremia ranges in humans [27], of the 4 DENV serotypes. In each vector competence experiment, mosquitoes were blood fed with a strain representing each of the 4 DENV serotypes at the 2 infectious doses. Ten individuals were then collected daily for 20 days, starting from the first day post-infection (DPI), to measure DENV infection status and infection kinetics in 3 key tissues—midgut (MG), carcass (CA) and salivary glands (SG)—per mosquito.

### The proportion of DENV-infected mosquitoes is affected by both infectious dose and DPI

**Fig 1:**
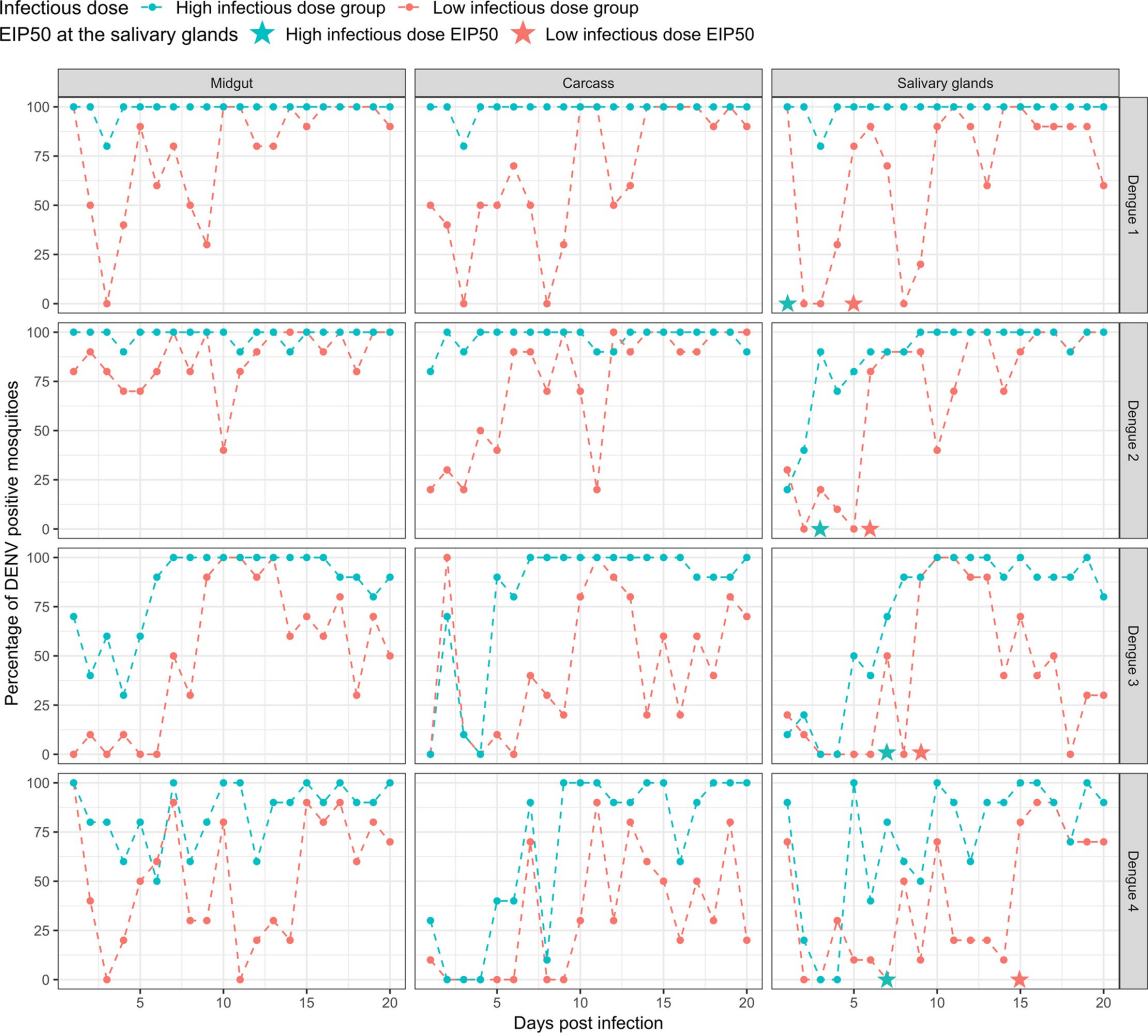
Susceptibility of DENV strains by DPI, tissue and infectious dose. The percentage of DENV infectious mosquitoes calculated daily out of 10 individuals. Low infectious dose in red and high infectious dose in blue. Stars represent EIP50 at the salivary gland for both infectious doses. Statistical analysis is presented in Table 2.

Infection status was determined by analyzing the proportion of individuals positive for DENV in each tissue at each DPI, based on the presence of a positive qRT-PCR. Infection prevalence (Fig 1) in the MG, CA and SG in all the serotypes was significantly influenced by the infectious dose and DPI (Table 1). In only one case was there a significant interaction between these 2 factors (Table 1, DENV-3 SG, GLM, F = 5.23, p = 0.02), indicating that, in general, dose and age of infection act independently to shape infection status.

**Table 1:** Susceptibility by Strain, Tissue, Dose and DPI.

*Serotype*	*Tissue*	*Factor*	*F*	*df*	*p-value*	*Significance*
DENV-1	MIDGUT	DOSE	29.79	38	<0.001	***
		DPI	19.38	37	<0.001	***
		DOSE: DPI	0.4	36	0.50	ns
	CARCASS	DOSE	49.33	38	<0.001	***
		DPI	30.68	37	<0.001	***
		DOSE: DPI	0.31	36	0.57	ns
	SALIVARY GLANDS	DOSE	31.78	38	<0.001	***
		DPI	12.09	37	0.001	**
		DOSE: DPI	0.40	36	0.52	ns
DENV-2	MIDGUT	DOSE	16.76	38	<0.001	***
		DPI	4.67	37	0.03	*
		DOSE: DPI	0.30	36	0.58	ns
	CARCASS	DOSE	15.86	38	<0.001	***
		DPI	7.49	37	0.001	**
		DOSE: DPI	0.12	36	0.72	ns
	SALIVARY GLANDS	DOSE	5.21	38	0.02	*
		DPI	30.71	37	<0.001	***
		DOSE: DPI	0.25	36	0.61	ns
DENV-3	MIDGUT	DOSE	16.28	38	<0.001	***
		DPI	18.52	37	<0.001	***
		DOSE: DPI	0.08	36	0.77	ns
	CARCASS	DOSE	15.48	35	<0.001	***
		DPI	6.24	34	0.01	*
		DOSE: DPI	1.04	33	0.31	ns
	SALIVARY GLANDS	DOSE	6.95	38	0.01	*
		DPI	13.72	37	<0.001	***
		DOSE: DPI	5.23	36	0.02	*
DENV-4	MIDGUT	DOSE	30.69	37	<0.001	***
		DPI	17.35	36	<0.001	***
		DOSE: DPI	1.47	35	0.23	ns
	CARCASS	DOSE	20.78	33	<0.001	***
		DPI	14.01	32	<0.001	***
		DOSE: DPI	0.66	34	0.42	ns
	SALIVARY GLANDS	DOSE	10.62	38	0.002	**
		DPI	18.07	37	<0.001	***
		DOSE: DPI	0.12	36	0.73	ns

*P<0.05

**P<0.01

***P<0.001.

ns. not significant.

Mosquito populations fed with higher doses had higher infection rates across tissues and strains (Fig 1). The DENV-1 and -2 strains exhibited a higher infection rate in all tissues and at both infectious doses compared to the DENV-3 and -4 strains. DENV-4 exhibited especially low infection rates in all tissues at the low infectious dose. The significance of DPI demonstrates that infection status is heavily time-dependent. For the DENV-1 and -2 strains, in the high infectious dose, infection rates were constantly high throughout 20 DPI in contrast to the low infectious dose, where there is noticeable difference between the infection rates between early and late DPI. For the DENV-3 strain, both infectious doses peaked in infection rate at ~12 days in all tissues. In the DENV-4 strain, infection levels are weaker than the other strains, which may contribute to the greater variation in the effect of time across tissues and doses.

### EIP is affected by the serotype strain and initial infectious dose

If we take SG detection of viral load as a proxy for presence of virus in the saliva, the susceptibility data (Fig 1) can also be used to shed light on EIP. EIP can be measured in multiple ways, including the day of first arrival of virus in the tissue/saliva or the point at which 50% of the population has infected tissue/saliva. For all strains and infectious doses, viral load can be detected in the SG already by day 2. In many cases, especially for low doses, viral load appears in the SG and then detection is lost for several days before returning and becoming stable (Fig 1). If we calculate an EIP_50_ for populations where infection continues for more than two days, we see that infectious dose and serotype strain diversity are predictors; for the strains representing DENV 1–4 at low-dose, they are 5, 6, 9 and 15 days, respectively, while for the high-dose they are 1, 3, 7 and 7 days.

### Intra-host DENV kinetics are influenced by initial infectious dose and strain

**Fig 2:**
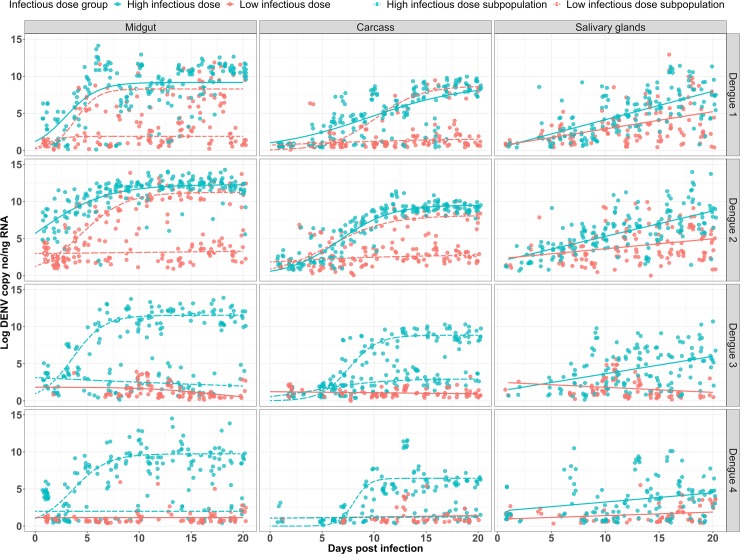
DENV kinetics by strain, tissue and infectious dose. For the midgut and carcass, lines represent the fitted parametric growth curves for DENV loads. Growth curves were fitted on logarithmic transformed data. For the salivary glands, lines represent a linear regression for DENV load. Linear regressions were fitted on logarithmic transformed data. For all tissues and serotypes, points represent individual mosquito values (n = 10); in red, low infectious dose (10^5^ DENV copies/ml); in blue, high infectious dose (10^8^ DENV copies/ml). Points for the subpopulations have been “jittered” to avoid overlap. Dashed lines represent subpopulations.

To quantify the kinetics of DENV infection, we assessed whether the cumulative change in DENV load in each tissue followed a trend that was either a sigmoidal or linear over time. Thus, we first fit a 3-parameter logistic model to DENV loads in each tissue, dose and serotype combination, which modeled the upper asymptote or the maximum DENV load, the slope or growth rate at the midpoint, and the midpoint or infection age which is halfway between the lower and upper asymptotes (i.e. ED50). We then estimated separate trends for each treatment combination. From the candidate models, the best fitting model for each treatment combination was selected using Akaike’s information criterion (AIC, S4 Table). For MG and CA tissues the best fitting model was the 3-parameter logistic model, while for the SG tissue we identified a linear relationship between DPI and DENV load. We used the slope and maximum DENV load parameters to compare the behavior of the different serotypes at different infectious doses and across tissues and times post-infection (Fig 2).

### Some treatments do not show substantial DENV replication and others exhibit subpopulation structure of DENV load in infected mosquitoes

For a number of treatments DENV loads did not rise to very high levels (Fig 2). For example, in the low-dose categories neither the DENV-3 nor -4 strains reached a load higher than log 10^3^ in any tissue as measured by population mean. In these cases, although we were able to fit a dose response curve (DRC) to the data, the parameters of max load and growth rate are not informative (S1 Table). Therefore, the subsequent discussion of growth parameter estimates focuses only on those treatments with substantial DENV loads and replication (Tables 2 and 3). For the remaining treatments we aimed to fit a single DRC to the different treatment combinations (infectious dose, tissue and strain). In several treatments, however, we identified potential subpopulation structure, with a proportion of the mosquitoes exhibiting high loads and the rest exhibiting very low loads. Those treatments were the low infectious dose in the MG and CA for strains of serotype DENV-1 and -2 and the high infectious dose in the MG and CA for strains of serotypes DENV-3 and -4 (Fig 2). After noticing subpopulation structure in our data, we applied Hartigan’s dip test for all datasets. For any with a significant result, we then used a bimodality coefficient test to partition the data. We found that using a coefficient of bimodality of >0.75 effectively split our datasets into two clear subpopulations (S2 Table) using the following criteria: (1) for the y-axis, we selected the lowest load in the histogram located between the two highest peaks (S1 Fig); and (2) for the x-axis, we identified the DPI where the two populations began to diverge.

**Table 2:** DRC parameters for successful infections by tissue, infectious dose and strain.

*Infectious dose*	*Serotype*	*Tissue*	*Parameter*	*Parameter estimate*	*Standard error*	*p-value*	*Significance*
HIGH	DENV-3	MIDGUT	Growth rate	0.66	0.07	<0.001	***
	DENV-1			0.64	0.21	0.003	**
	DENV-4			0.59	0.11	<0.001	***
	DENV-2			0.31	0.04	<0.001	***
	DENV-4	CARCASS		1.36	0.48	0.005	**
	DENV-3			0.73	0.1	<0.001	***
	DENV-2			0.4	0.03	<0.001	***
	DENV-1			0.21	0.05	<0.001	***
	DENV-2	MIDGUT	Max DENV load	12.2	0.2	<0.001	***
	DENV-3			11.5	0.17	<0.001	***
	DENV-4			9.74	0.26	<0.001	***
	DENV-1			9.11	0.27	<0.001	***
	DENV-2	CARCASS		9.53	0.16	<0.001	***
	DENV-1			8.99	1.24	<0.001	***
	DENV-3			8.85	0.19	<0.001	***
	DENV-4			6.44	0.21	<0.001	***
	DENV-1	MIDGUT	ED50	3.6	0.38	<0.001	***
	DENV-2			3.6	0.43	0.28	
	DENV-3			2.94	0.19	<0.001	***
	DENV-4			0.46	0.31	<0.001	***
	DENV-1	CARCASS		9.61	1.72	<0.001	***
	DENV-4			8.06	0.32	<0.001	***
	DENV-3			7.56	0.18	<0.001	***
	DENV-2			6.69	0.19	<0.001	***
LOW	DENV-1	MIDGUT	Growth rate	0.92	0.27	0.001	***
	DENV-2			0.46	0.05	<0.001	***
	DENV-1	CARCASS		0.45	0.07	<0.001	***
	DENV-2			0.36	0.08	<0.001	***
	DENV-2	MIDGUT	Max DENV load	11.24	0.27	<0.001	***
	DENV-1			8.28	0.27	<0.001	***
	DENV-1	CARCASS		8.7	0.44	<0.001	***
	DENV-2			8.1	0.37	<0.001	***
	DENV-2	MIDGUT	ED50	4.44	0.26	<0.001	***
	DENV-1			3.88	0.33	<0.001	***
	DENV-1	CARCASS		10.1	0.53	<0.001	***
	DENV-2			5.77	0.69	<0.001	***

*P<0.05

**P<0.01

***P<0.001. p-value indicates if parameter estimate is different than zero.

Standard error of parameter estimates. Treatments ranked by parameter estimate.

**Table 3:** DRC parameter contrasts for successful infections between strains, infectious dose and subpopulations.

*Infectious dose*	*Serotype contrast*	*Tissue*	*Parameter estimate*	*Standard error*	*p-value*	*Significance*
HIGH	DENV-1 vs DENV 2	MIDGUT	Growth rate	0.18	0.08	ns
			Max DENV load	0.36	<0.001	***
		CARCASS	Growth rate	0.05	0.001	**
			Max DENV load	1.02	0.58	ns
LOW		MIDGUT	Growth rate	0.25	0.07	ns
			Max DENV load	0.39	<0.001	***
		CARCASS	Growth rate	0.11	0.42	ns
			Max DENV load	0.59	0.31	ns
HIGH	DENV-1 vs DENV-3	MIDGUT	Growth rate	0.20	0.94	ns
			Max DENV load	0.34	<0.001	***
		CARCASS	Growth rate	0.13	<0.001	***
			Max DENV load	1.00	0.88	ns
HIGH	DENV-1 vs DENV-4	MIDGUT	Growth rate	0.20	0.78	ns
			Max DENV load	0.33	0.08	ns
		CARCASS	Growth rate	0.46	0.01	**
			Max DENV load	0.99	0.01	**
HIGH	DENV-2 vs DENV-3	MIDGUT	Growth rate	0.13	0.01	**
			Max DENV load	0.33	0.05	ns
		CARCASS	Growth rate	0.13	0.01	**
			Max DENV load	0.37	0.04	**
HIGH	DENV-2 vs DENV-4	MIDGUT	Growth rate	0.13	0.03	**
			Max DENV load	0.30	<0.001	***
		CARCASS	Growth rate	0.46	0.04	***
			Max DENV load	0.30	<0.001	***
HIGH	DENV-3 vs DENV-4	MIDGUT	Growth rate	0.14	0.63	ns
			Max DENV load	0.32	<0.001	***
		CARCASS	Growth rate	0.43	0.15	ns
			Max DENV load	0.29	<0.001	***

*P<0.05

**P<0.01

***P<0.001

ns, not significant.

### DENV load is always higher in the midgut than other tissues for high infectious dose

**Fig 3:**
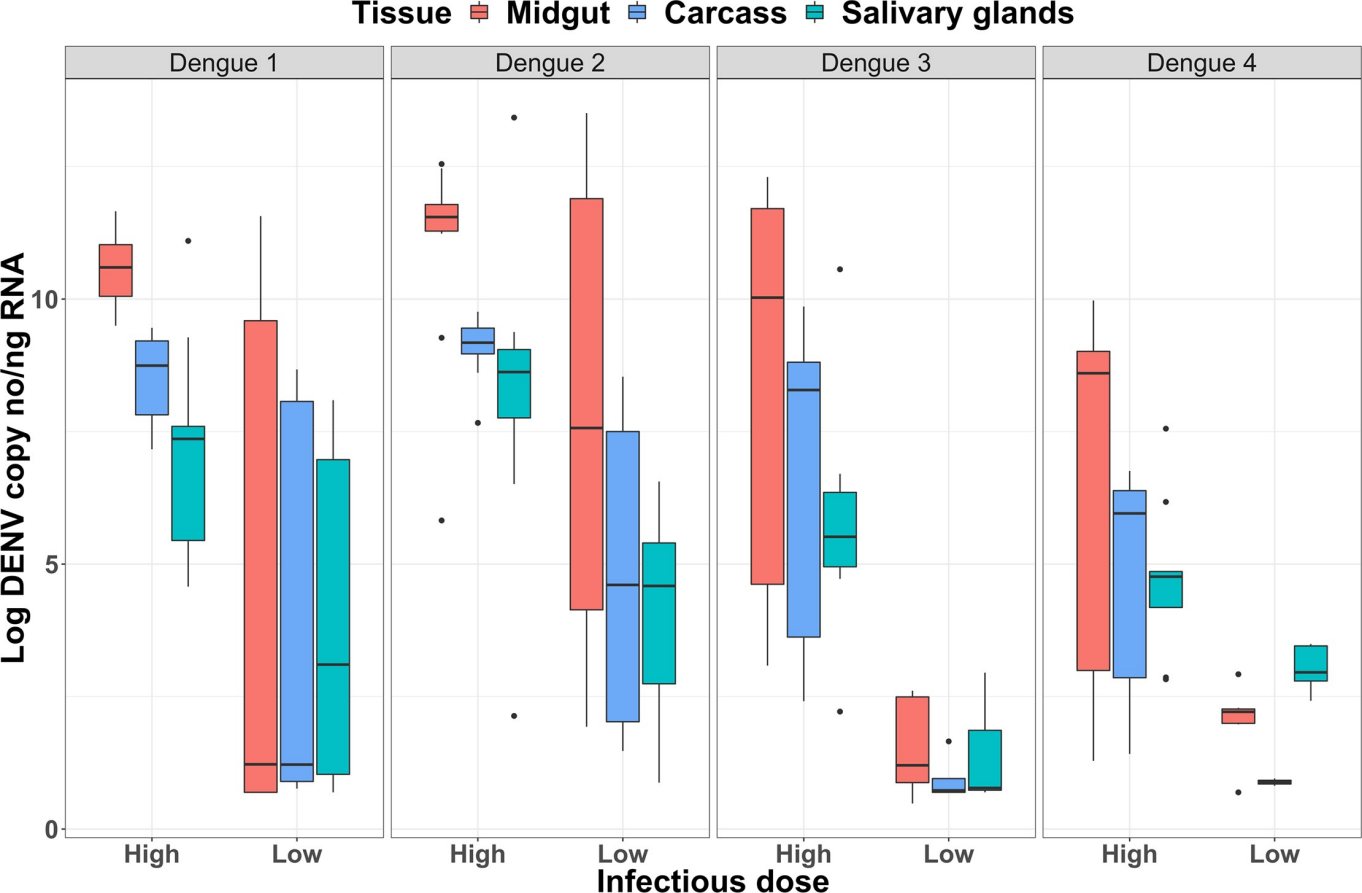
“Trickledown” or declining viral load with tissue progression. Average 20 DPI DENV load for the 4 dengue serotypes across infectious dose and tissues. Box plots show median values (**horizontal line in the box)**, 25–75% interquartile range (**upper–lower limits of the box**), 95% range of data (**error lines**).

In all serotypes, maximum DENV loads were consistently higher in the MG than the CA. Because we fit linear models to the SG data, we examined the average DENV load at the last DPI (20 days) in lieu of a maximum load (S2 Fig). For all serotypes and high infectious doses, the SG load at this point was lower than the CA loads. This strong pattern suggests a “trickledown” effect for DENV load across the sequentially infected tissues (Fig 3). In contrast, low infectious doses resulted in low DENV loads in all tissues and for all strains, and DENV-3 and -4 could not be evaluated due to their lack of successful infection and failure of the selected model to run. Additionally, we ran generalized linearized models on DENV load in the MG and CA (for high dose only) as a predictor for the SG DENV load. We found no significant predictive ability regardless of the serotype and tissue (S5 Table).

### Tissue-specific DENV kinetics vary by strain in the high infectious dose

In the MG, the maximum loads of DENV-2 (12.2 log c/ng) and -3 (11.5 log c/ng) are similarly high and greater than the strains representing serotypes 1 (9.11 log c/ng) and 4 (9.74 log c/ng) (Table 2). In the CA, the DENV-1 (8.99 log c/ng) and -2 (9.53 log c/ng) strains have the highest loads and are both significantly different from DENV-3 (8.85 log c/ng) and -4 (6.44 log c/ng) strains (Table 3). In the SG, the average final load (DPI 20) for the DENV-2 strain is not different from the DENV-1 and -3 strains but is greater than the DENV-4 (S2 Fig). Although we have examined only one representative strain from each serotype, these patterns suggest that DENV-2 may be better at replicating in mosquitoes and that the other serotypes do not show consistent patterns of dominance over one another across tissues. For growth rate, no one strain appeared to dominate over the others across multiple tissues (Tables 3 and 4). For example, the DENV-3 strain has the highest growth rate (change in viral copies/day) in the MG (0.66 log DENV copies per DPI), whereas DENV-2 strain has the lowest (0.31 log DENV copies per DPI). In the CA, DENV-4 has the highest growth rate (1.36 log DENV copies per DPI), whereas DENV-1 has the lowest (0.21 log DENV copies per DPI). Interestingly, in the SG all strains have distinctly different growth rates, ranked in the following order: DENV-1, 2, 3, and 4 (Table 4). This suggests that the growth rates in early process/upstream tissues are not predictive of rates in the final tissue controlling transmissibility.

### The DENV-2 strain has a higher maximum DENV load than the DENV-1 strain in the low infectious dose

For the low infectious dose, we only considered mosquitoes infected with either DENV-1 or -2 strains, as the remaining strains did not exhibit “successful” infections meaning that even though infection rate was high, the actual DENV load was substantially low. Here, we saw that the DENV-2 strain had a significantly higher max DENV load in the MG and a higher average final load in the SG than the DENV-1 strain. For growth rate, there were no differences between the DENV-1 and DENV-2 strains in the MG or CA, but in SG, the DENV-1 strain was significantly higher (Table 5).

**Table 4:** Linear regression model for SG.

*Infectious dose*	*Serotype*	*Term*	*Slope*	*Standard error*	*F*	*p-value*	Significance
LOW	DENV-1	DPI	0.23	0.03	6.12	<0.01	**
	DENV-2	DPI	0.13	0.03	3.51	<0.01	**
	DENV-3	DPI	−0.06	0.03	-1.81	0.073	ns
	DENV-4	DPI	0.04	0.02	2.31	0.023	*
HIGH	DENV-1	DPI	0.38	0.03	10.98	<0.01	**
	DENV-2	DPI	0.34	0.03	11.38	<0.01	**
	DENV-3	DPI	0.23	0.04	5.24	<0.01	**
	DENV-4	DPI	0.12	0.04	2.87	<0.01	**

*P<0.05

**P<0.01

***P<0.001. Tukey for contrasts.

ns, not significant.

**Table 5:** Serotype contrasts for growth rate in SG.

*Infectious dose*	*Serotype contrast*	*Standard error*	*Df*	*p-value*	Significance
LOW	DENV-1 –DENV-2	0.16	1026	0.24	ns
	DENV-1 –DENV-3	1.81	1026	< .0001	***
	DENV-1 –DENV-4	2.14	1026	< .0001	***
	DENV-2 –DENV-3	2.95	1026	< .0001	***
	DENV-2 –DENV-4	3.49	1026	< .0001	***
	DENV-3 –DENV-4	0.40	1026	0.97	ns
HIGH	DENV-1 –DENV-2	0.08	1026	0.0001	***
	DENV-1 –DENV-3	0.58	1026	0.004	**
	DENV-1 –DENV-4	1.15	1026	< .0001	***
	DENV-2 –DENV-3	1.64	1026	< .0001	***
	DENV-2 –DENV-4	3.27	1026	< .0001	***
	DENV-3 –DENV-4	0.51	1026	0.054	ns

*P<0.05

**P<0.01

***P<0.001. Tukey for contrasts.

ns, not significant.

## Discussion

To explore the role of infectious dose, serotype and tissue in viral infection kinetics we sampled DENV loads in populations of infected mosquitoes over numerous, sequential time-points of the mosquito’s lifespan relevant to transmission in the field. We reveal that the kinetics of DENV infection in the midgut, carcass and salivary glands of the mosquito *Aedes aegypti* are strikingly different among the strains selected for this study, and that these differences are also driven by the initial infectious dose. Specifically, we showed that (1) initial infectious dose dictates infection frequency, with the DENV-1 and -2 strains analyzed here infecting a greater proportion of mosquitoes than the DENV-3 and -4 strains; (2) for DENV-3 and -4 strains only, the high infectious dose led to strong infection, with lower doses producing lower viral replication; (3) viral growth rates in particular tissues did not predict the total DENV load in that same tissue, with some strains having higher growth rate but lower total DENV load; (4) in the salivary glands all strains have independent growth rates not predicted by growth rates in previous tissues; and (5) for every tissue and infectious dose, the DENV-2 strain reached a higher viral load according to both the average final load at 20 DPI and max DENV load.

The initial infectious dose has been shown to influence the likelihood of a mosquito becoming infected following an infectious blood meal and whether there is ultimately dissemination to the salivary glands after a blood meal [28, 29]. The amount of virus in human blood required to infect ~50% of a mosquito population varies between serotypes, with DENV-1 and -2 requiring ~10-fold less than DENV-3 and -4. This same rank order is observed here. However, strain variation within each serotype is substantial [30], so further comparisons across multiple strains within each serotype are clearly needed to draw any conclusions about whether the strains we have selected are predictive of the entire serotype.

We were also able to identify a potential threshold of virus needed in the blood meal to initiate successful infections and assure likely transmissibility via the salivary glands. A similar threshold effect has been observed in *Drosophila melanogaster* populations challenged with systemic bacterial infections [31].The study identified two possible outcomes of infection; (1) where hosts died with high bacterial burden or (2) where hosts survived with chronic infection and low pathogen load. Bacterial load and the time it took the host to establish an immune response were found to determine the trajectory of infection. Similarly, we saw either high infection (leading to transmissibility) or low/loss of infection. Interestingly, for our study, the threshold effect was driven by the initial infectious dose and by serotype. DENV-1 and -2 strains required less virus to establish an infection, whereas, DENV-3 and -4 strains required substantially more. The failure to initiate a successful infection could be due to poor replication in the midgut or the midgut preventing dissemination [10, 11, 32]. This pattern is not unique to the midgut, however, with low and inefficient replication also in disseminated tissues, such as the carcass. The data may simply be the result of low population sizes of virus in combination with the large number of vacant cellular niches. During infection DENV selectively annexes and manipulates host metabolism and machinery to increase viral translation and replication [33–35], while also enhancing replication of mosquito cells to increase niche availability in the mosquito [36]. The more virions that infect a given cell, the more efficient this process becomes and, thus, more viruses are produced. Initial viral load combined with inherent replicative differences between strains is likely to produce the pattern we see in our data, that the DENV-1 and 2 strains require a lower dose to produce successful infections.

Interestingly, higher viral growth rates in tissues did not necessarily lead to higher DENV loads. For example, for the high infectious dose of DENV-1, the midgut had the highest max DENV load compared to the carcass, however its growth rate was smaller. This relationship was seen for the other treatments as well. It is possible that the migration of viruses between tissues via the tracheal system or hemolymph [37] or directly from nearby tissues is impacting the total viral counts. In support of tracheal or hemolymph travel is the initial spike in infection rate in salivary glands followed by a reduction to zero and then a second front of infection several days later. It is possible that the latter wave represents the larger migration of virus from bodily tissues that then establishes permanent infection. Given how the circulatory system of the mosquito works, viral particles could be carried from tissues that are not in immediate proximity to the salivary glands. This process could directly affect growth rate in upstream tissues. The salivary glands also represent a special case of the entry and exit balance, with virus being excreted into the saliva and hence lost from tissue measures. Under a more natural setting additional factors would likely affect kinetics including, exposure to non-infectious blood meals prior to consumption of an infectious [38], consumption of sequential dengue infectious meals [39] or interactions between DENV and other viruses present in blood meals or the mosquito body [40].

The disconnect between kinetics in intermediate tissues and those of the salivary glands suggests that there are either stochastic processes driving kinetics in early tissues that do not predict how many viruses make it to the salivary glands, or that factors affecting replication rate in the salivary glands are somewhat independent [23]. For example, previous research has demonstrated that the strength and efficacy of immunity varies across tissues [23] and in response to different serotypes [41]. It is also possible that DENV is modulating the vector immune response in a strain- or tissue-dependent manner [42]. Differences between the salivary glands and other tissues may also be due to the number of cellular niches, and/or unique replication rates in those niches. For example, in contrast to the midgut epithelium, DENV tropism in the salivary glands appears to be heterogenous within the tissue; this might be due to the distribution of cellular receptors [43], with a possible higher concentration of viral entry receptors in the lateral distal lobes of the tissue [44]. In future studies, it would be useful to quantify the production rate of virus using negative strand strain-specific PCR methods [45] in the midgut and in the salivary glands relative to the availability of cellular niches.

The differences in viral kinetics between the DENV strains analyzed here, particularly in the salivary glands, might explain disparities in transmission potential in the field. A range of studies have linked different genotypes to overall dominance in terms of circulation in human populations both at local and global scales [46–49]. Additionally, DENV variants with a replicative advantage in both the host and vector can spread more rapidly, eventually displacing those with lower fitness [21]. Our findings indicate that 2 of the 4 DENV strains studied here show greater capacity to replicate in the mosquito. In some cases, these differences may have consequences for public health, as some specific variants of DENV have been linked to more severe clinical manifestations than others [50–53]. These findings also have implications for the mosquito’s contribution to the DENV epidemiological landscape, with the understanding that aspects of human immunity, including cross-reactivity and non-specific serotype responses also shape the nature of circulating variants [54]. Lastly, our work provides better insight into ideal design of vector competence experiments and the selection of gene candidates for the engineering of virus refractory mosquitoes. Specifically, our findings support a greater focus on salivary gland or saliva-based measures of infection and genes that either completely reduce viral loads in the midgut or that control viral replication in the endpoint tissue, salivary glands.

## Materials and methods

### *Ae*. *aegypti* rearing and virus preparation

Approximately 8000 eggs of an *Ae*. *aegypti* inbred line collected in Townsville, Australia 13 generations previously were vacuum hatched and divided into individual 30 × 40 × 8-cm plastic trays containing 1 liter of reverse osmosis (RO) autoclaved water. Larvae (~150) were placed in individual trays containing 3 liters of RO autoclaved water, supplemented with common fish food (Tetramin®, Melle, Germany). Pupae were collected and placed into breeding cages, containing approximately 450 mosquitoes each. Adults were provided 10% sucrose *ad libitum*. All mosquitoes were reared in a controlled environment at 26°C, 75% relative humidity and a 12-hr light/dark cycle.

The DENV serotypes/strains used for this experiment are listed in Table 6. The virus was propagated in cell culture, as described previously [55]. *Ae*. *albopictus* C6/36 cells were grown at 26°C in RPMI 1640 medium (Invitrogen, Carlsbad, CA) supplemented with 10% fetal bovine serum (FBS), 1× Glutamax (Invitrogen) and HEPES buffer. Cells were first allowed to form monolayers of around 60–80% confluence in T175 flasks (Sigma Aldrich, St. Louis, MO), and then inoculated with DENV and maintained in RPMI medium supplemented with 2% FBS. After 7 days post-inoculation, live virus was harvested, titrated via absolute quantification PCR and adjusted to two final viral loads of 10^5^ and 10^8^ DENV copies per ml. Single-use aliquots were stored at −80°C for subsequent plaque assay titration.

**Table 6:** The DENV strains used in this study.

*Serotype*	*Strain*	*Passage*	*GenBank accession number*	*Place of origin*	*Collection date*
DENV-1	FR-50	10	FJ432734.1	Vietnam	2007
DENV-2	ET-300	11	EF440433.1	East Timor	2000
DENV-3	Cairns/08/09	9	JN406515.1	Australia	2008
DENV-4	33–188	12	None	Vietnam	NA

Na; not available.

### Mosquito infections

Prior to infection, female mosquitoes were sorted and placed in 1-liter plastic cups with a density of ~150 individuals. Sucrose was removed from the mosquitoes 24 hrs, prior to oral infection. Double-chamber glass feeders were covered with pig intestine previously immersed in a 10% sucrose solution. Water heated to 37°C was circulated in the outer chamber of the feeders, and a 1:1 mix of defibrinated sheep blood and the previously titrated DENV virus was placed inside the feeder. The mosquitoes were split into 2 feeding groups, each allowed to feed for ~2 hrs. One group was fed with a DENV infectious blood meal concentration of 10^8^ and the other with 10^5^ DENV copies/ml. After 24 hrs, blood fed mosquitoes were identified by visual inspection and separated into cups of 10 individuals.

### Mosquito dissection and RNA extraction

Blood fed *Ae*. *aegypti* mosquitoes were collected daily, 10 per feeding group, over 20 days. Mosquitoes were anesthetized by chilling and dissected on a chill plate in a drop of sterile phosphate buffer saline (PBS). Midgut, salivary glands and carcass tissues were placed in separated 1.5-ml microcentrifuge tubes (Sarstedt, Nümbrecht, Germany) containing 200 μl of TRIzol reagent (Invitrogen, Carlsbad, CA, USA) and 2-mm glass beads. Samples were homogenized and frozen at −80°C. RNA extraction was performed using the TRIzol RNA extraction protocol according to the manufacturer’s instructions, and RNA was eluted in 25 μl of nucleic acid-free water. Samples were DNase treated (Life Technologies, Carlsbad, CA, USA) according to the manufacturer instructions [56]. Total RNA was determined with a NanoDrop lite spectrophotometer (Thermo Scientific, Waltham, MA).

### DENV absolute quantification via qRT-qPCR

Quantitative real-time PCR (qRT-PCR) reactions for DENV detections were performed with 4× TaqMan fast virus 1-step master mix (Roche Applied Science, Switzerland), PCR-grade water, 10 μM of DENV primers and probe and 2.5 μl of RNA in a final volume of 10 μl. Reactions were run in microseal 96 microplates (Life Technologies Life, Technologies, Carlsbad, CA) covered with optically clear film. LightCycler 480 (Roche Applied Science, Switzerland) thermal cycling conditions were 50°C for 5 min for reverse transcription, 95°C for 10 s for RT inactivation/denaturation followed by 50 amplification cycles of 95°C for 3 s, 60°C 30 s and 72°C for 1 s.

Standard curves were generated from triplicate samples on each plate spanning the range of 10 to 10^8^ copies/reaction of DENV fragment copies. DENV standards were constructed as described elsewhere [57], and they contained the 3’UTR of the DENV genome. The limit of detection was set at 10 copies in this experiment; water was used as a negative control and standards were run in triplicates. The concentration of DENV genome copies in each sample was extrapolated from the standard curve as DENV copies per nanogram of total RNA.

The primer sequences used for the detection of DENV, as previously used [58], were F: 5′-AAGGACTAGAGGTTAGAGGAGACCC-3′, R: 5′CGTTCTGTGCCTGGAATGATG-3′ and P: 5′-HEX-AACAGCATATTGACGCTGGGAGAGACCAGA-BHQ1-3′.

### Survival assay

A second set of Townsville mosquitoes were reared in the same conditions as above. Mosquitoes were blood fed with the two viral concentrations as above (10^8^ and 10^5^ DENV copies/ml). A total of 80 fed mosquitoes per concentration were separated into clear 200-ml cups, each containing 10 individuals. They were monitored daily to assess death until all had expired. Neither DENV serotypes nor infectious doses had an effect on survival compared to a blood-only fed control (S3 Fig, S3 Table).

### Data analysis

Data analysis was carried out in R v 3.6.0 (http://www.r-project.org/). For statistical analysis of the infection frequency, quasibinomial GLM was used to correct for overdispersion of data, and Tukey for contrasts was used for *post hoc* comparisons. For the kinetics of DENV virus, first we fit a 3-parameter logistic DRC model to DENV loads in each tissue, dose and serotype combination, which modeled the upper asymptote or the maximum DENV load, the slope or growth rate at the midpoint, and the midpoint or infection age which is halfway between the lower and upper asymptotes (i.e. ED50). We then estimated separate trends for each treatment combination. From the candidate models, the best fitting model for each treatment combination was selected using Akaike’s information criterion (AIC). For MG and CA tissues the best fitting model was the 3-parameter logistic model, while for the SG tissue we identified a linear relationship between DPI and DENV load. DRC parameters and pairwise comparisons were estimated with the DRC package [59]. The best model was selected based on the AIC (yielding the lowest) values. In general, for the salivary glands, the best fit was a linear model; thus, they were subsequently analyzed in this fashion. For the average DENV load at DPI 20, significant differences were based on Tukey *post hoc* comparison following ANOVAs. All DENV loads were reported on a log scale given the value range.

## Supporting information

S1 Table DRC estimates for unsuccessful infections by tissue, infectious dose and DENV strain. (DOCX)

S2 Table Multimodal distribution analysis. (DOCX)

S3 Table Survival curve statistical analysis. (DOCX)

S4 Table AIC values for the DRC model. (DOCX)

S5 Table Results of the GLM fitted to salivary glands DENV load. (DOCX)

S1 Fig Density plots. Dotted line indicates data split point.
(DOCX)

S2 Fig Average salivary gland DENV load at the last DPI (20 days). Average DENV load at DPI 20 for all 4 DENV serotypes at 2 infectious doses (High: 1 × 10^8^, low: 1 × 10^5^ DENV copies/ml). All box plots show median and interquartile ranges (n = 10 per treatment). Significant differences are based on Tukey *post hoc* comparison following ANOVAs on log-transformed data. Only significant differences are shown. *p<0.05.
(DOCX)

S3 Fig Survival curves for all treatments. Survival curve analysis of control and the 4 DENV serotypes at 2 infectious doses (High: 1 × 10^8^, low: 1 × 10^5^ DENV copies/ml). There was no significant difference between treatments (χ^2^ = 12.9, df = 8, p = 0.1). Dotted outside lines represent 95% confidence intervals. Each line at the top represents individual treatments.
(DOCX)
