# Supplementary material for: Intra-host growth kinetics of dengue virus in the mosquito Aedes aegypti
Source: PLoS Pathog. 2019 Dec 2;15(12):e1008218. doi: 10.1371/journal.ppat.1008218 (PMC6907869; doi:10.1371/journal.ppat.1008218)
Supplement: S1 Table — (DOCX) [file ppat.1008218.s001.docx]

**Supplemental Table 1. DRC estimates for unsuccessful infections by tissue, infectious dose and DENV strain**

| Infectious dose | Serotype | Tissue | Subpopulation threshold | Parameter | Estimate | SE | p-value | Significance |
| --- | --- | --- | --- | --- | --- | --- | --- | --- |
| LOW | DENV-1 | MIDGUT | Unsuccessful | Growth rate | 1.25 | 0.81 | 0.12 |  |
|  |  |  |  | Max DENV load | 1.96 | 0.14 | <0.001 | *** |
|  |  |  |  | ED50 | 1.14 | 0.69 | 0.1 |  |
|  |  | CARCASS |  | Growth rate | 0.13 | 0.15 | 0.38 |  |
|  |  |  |  | Max DENV load | 1.64 | 0.58 | 0.001 |  |
|  |  |  |  | ED50 | 1.91 | 5.16 | 0.71 |  |
|  | DENV-2 | MIDGUT |  | Growth rate | 0 | 0.03 | 0.79 |  |
|  |  |  |  | Max DENV load | 6.56 | 28.63 | 0.81 |  |
|  |  |  |  | ED50 | 1.14 | 0.68 | 1.67 |  |
|  |  | CARCASS |  | Growth rate | 0.04 | NA | NA |  |
|  |  |  |  | Max DENV load | 4.04 | NA | NA |  |
|  |  |  |  | ED50 | 0 | NA | NA |  |
| HIGH | DENV-3 | MIDGUT |  | Growth rate | -0.03 | 0.03 | 0.93 |  |
|  |  |  |  | Max DENV load | 6.22 | 9.66 | 0.64 |  |
|  |  |  |  | ED50 | 0 | 78.7 | 1 |  |
| LOW | DENV-3 |  | NTA | Growth rate | -0.3 | 0.17 | 0.08 |  |
|  |  |  |  | Max DENV load | 1.85 | 0.26 | <0.001 | *** |
|  |  |  |  | ED50 | 17.1 | 1.84 | <0.001 | *** |
| HIGH | DENV-3 | CARCASS | Unsuccessful | Growth rate | 0.31 | 0.13 | 0.02 |  |
|  |  |  |  | Max DENV load | 2.96 | 0.28 | <0.001 | *** |
|  |  |  |  | ED50 | 4.56 | 1.09 | <0.001 | *** |
| LOW | DENV-3 |  | NTA | Growth rate | -0.02 | 0.03 | 0.49 |  |
|  |  |  |  | Max DENV load | 2.44 | 4.99 | 0.62 |  |
|  |  |  |  | ED50 | 0 | 190.7 | 1 |  |
| HIGH | DENV-4 | MIDGUT | Unsuccessful | Growth rate | -0.04 | 0.03 | 0.1552 |  |
|  |  |  |  | Max DENV load | 4.93 | 0.19 | <0.001 | *** |
|  |  |  |  | ED50 | 0 | 50.3 | 1 |  |
| LOW | DENV-4 |  | NTA | Growth rate | 0.15 | 0.3 | 0.6 |  |
|  |  |  |  | Max DENV load | 1.38 | 0.52 | 0.01 | * |
|  |  |  |  | ED50 | 0 | 4.55 | 1 |  |
| HIGH | DENV-4 | CARCASS | Unsuccessful | Growth rate | 0.02 | 0.06 | 0.74 |  |
|  |  |  |  | Max DENV load | 3.63 | 20.24 | 0.85 |  |
|  |  |  |  | ED50 | 17.12 | 745.6 | 0.9 |  |
| LOW | DENV-4 |  | NTA | Growth rate | 0.64 | 0.43 | 0.14 |  |
|  |  |  |  | Max DENV load | 1.31 | 0.16 | <0.001 | *** |
|  |  |  |  | ED50 | 7.25 | 2.17 | 0.001 | ** |
| *NA, not available due to poor fitting to the log logistic model. *NTA no threshold applied. *P<0.05, **P<0.01, ***P<0.001. SE of the estimate, p-value indicates if estimate is different than zero. | | | | | | | | |

|  |  |
| --- | --- |
