# Supplementary material for: Intra-host growth kinetics of dengue virus in the mosquito Aedes aegypti
Source: PLoS Pathog. 2019 Dec 2;15(12):e1008218. doi: 10.1371/journal.ppat.1008218 (PMC6907869; doi:10.1371/journal.ppat.1008218)
Supplement: S2 Table — (DOCX) [file ppat.1008218.s002.docx]

**Supplemental Table 2. Multimodal distribution analysis**

| Treatments | Hartigan’s dip test | Bimodality coefficient |
| --- | --- | --- |
| DENV-1 CA LOW | <2.2e-16 | 0.81 |
| DENV-1 MG LOW | 2.60E-05 | 0.86 |
| DENV-2 CA LOW | 0.001386 | 0.72 |
| DENV-2 MG LOW | 4.24E-05 | 0.79 |
| DENV-3 CA HIGH | 7.32E-06 | 0.73 |
| DENV-3 MG HIGH | <2.2e-16 | 0.75 |
| DENV-4 CA HIGH | 5.18E-06 | 0.89 |
| DENV-4 MG HIGH | <2.2e-16 | 0.93 |
| DENV-1 CA HIGH | 7.83E-06 | 0.67 |
| DENV-1 MG HIGH | 0.5921 | 0.63 |
| DENV-2 CA HIGH | 0.0001161 | 0.75 |
| DENV-2 MG HIGH | 0.9904 | 0.63 |
| If the p value is >0.05, it suggests bimodality. | |  |
