# Supplementary material for: Intra-host growth kinetics of dengue virus in the mosquito Aedes aegypti
Source: PLoS Pathog. 2019 Dec 2;15(12):e1008218. doi: 10.1371/journal.ppat.1008218 (PMC6907869; doi:10.1371/journal.ppat.1008218)
Supplement: S3 Table — (DOCX) [file ppat.1008218.s003.docx]

**Supplemental Table 3. Survival curve statistical analysis**

| *Treatment* | *N* | *Observed* | *Expected* | *(O-E)^2/E* | *(O-E)^2/V* |
| --- | --- | --- | --- | --- | --- |
| Control | 80 | 80 | 88.5 | 0.82 | 1.06 |
| DENV-1 High | 80 | 80 | 98.7 | 3.52 | 4.93 |
| DENV-1 Low | 80 | 80 | 75.2 | 0.31 | 0.39 |
| DENV-2 High | 80 | 80 | 64.7 | 3.62 | 4.44 |
| DENV-2 Low | 80 | 80 | 86.4 | 0.47 | 0.60 |
| DENV-3 High | 80 | 80 | 81.1 | 0.02 | 0.03 |
| DENV-3 Low | 80 | 80 | 82.1 | 0.05 | 0.07 |
| DENV-4 High | 80 | 80 | 68.7 | 1.85 | 2.29 |
| DENV-4 Low | 80 | 80 | 74.3 | 0.43 | 0.54 |
| Chisq = 12.9 on 8 degrees of freedom, p= 0.01. | | | | |  |
