# Supplementary material for: Intra-host growth kinetics of dengue virus in the mosquito Aedes aegypti
Source: PLoS Pathog. 2019 Dec 2;15(12):e1008218. doi: 10.1371/journal.ppat.1008218 (PMC6907869; doi:10.1371/journal.ppat.1008218)
Supplement: S4 Table — (DOCX) [file ppat.1008218.s004.docx]

**Supplemental Table 4. AIC values for the DRC model**

| Serotype | Dose | Tissue | Subpopulation threshold | AIC |
| --- | --- | --- | --- | --- |
| DENV-1 | High | MG | NA | 967 |
| DENV-1 | Low | MG | Successful | 318 |
| DENV-1 | Low | MG | Unsuccessful | 341 |
| DENV-2 | High | MG | NA | 775 |
| DENV-2 | Low | MG | Successful | 386 |
| DENV-2 | Low | MG | Unsuccessful | 360 |
| DENV-3 | High | MG | Successful | 392 |
| DENV-3 | High | MG | Unsuccessful | 306 |
| DENV-3 | Low | MG | NA | 263 |
| DENV-4 | High | MG | Successful | 605 |
| DENV-4 | High | MG | Unsuccessful | 228 |
| DENV-4 | Low | MG | NA | 282 |
| DENV-1 | High | CA | NA | 786 |
| DENV-1 | Low | CA | Successful | 181 |
| DENV-1 | Low | CA | Unsuccessful | 300 |
| DENV-2 | High | CA | NA | 621 |
| DENV-2 | Low | CA | Successful | 199 |
| DENV-2 | Low | CA | Unsuccessful | 340 |
| DENV-3 | High | CA | Successful | 340 |
| DENV-3 | High | CA | Unsuccessful | 301 |
| DENV-3 | Low | CA | NA | 160 |
| DENV-4 | High | CA | Successful | 362 |
| DENV-4 | High | MG | Unsuccessful | 163 |
| DENV-4 | Low | MG | NA | 189 |
