# Supplementary material for: Intra-host growth kinetics of dengue virus in the mosquito Aedes aegypti
Source: PLoS Pathog. 2019 Dec 2;15(12):e1008218. doi: 10.1371/journal.ppat.1008218 (PMC6907869; doi:10.1371/journal.ppat.1008218)
Supplement: S5 Table — (DOCX) [file ppat.1008218.s005.docx]

**Supplemental Table 5. Results of the GLM fitted to salivary glands DENV load**

|  | Estimate | SE | t-value | P=value |
| --- | --- | --- | --- | --- |
| Intercept | 1.06 | 2.36 | 0.44 | 0.66 |
| DENGUE-2 | 41.94 | 75.43 | 0.55 | 0.58 |
| DENGUE-3 | 0.14 | 2.41 | 0.05 | 0.95 |
| DENGUE-4 | -29.65 | 20.55 | -1.44 | 0.16 |
| Carcass load | 0.13 | 0.29 | 0.44 | 0.66 |
| Midgut load | 0.05 | 0.24 | 0.21 | 0.83 |
| DENGUE-2:Carcass load | -4.69 | 8.34 | -0.56 | 0.58 |
| DENGUE-3:Carcass load | -0.03 | 0.30 | -0.13 | 0.89 |
| DENGUE-4:Carcass load | 6.15 | 4.24 | 1.44 | 0.16 |
| DENGUE-2:Midgut load | -3.50 | 6.34 | -0.55 | 0.58 |
| DENGUE-3:Midgut load | -0.01 | 0.24 | -0.07 | 0.94 |
| DENGUE-4:Midgut load | 3.02 | 2.09 | 1.44 | 0.16 |
| Carcass load:Midgut load | -0.01 | 0.03 | -0.33 | 0.73 |
| DENGUE-2:Carcass load:Midgut load | 0.39 | 0.70 | 0.56 | 0.58 |
| DENGUE-3:Carcass load:Midgut load | 0.008 | 0.03 | 0.27 | 0.78 |
| DENGUE-4:Carcass load:Midgut load | -0.62 | 0.42 | -1.45 | 0.16 |
