# Supplementary material for: Intra-host growth kinetics of dengue virus in the mosquito Aedes aegypti
Source: PLoS Pathog. 2019 Dec 2;15(12):e1008218. doi: 10.1371/journal.ppat.1008218 (PMC6907869; doi:10.1371/journal.ppat.1008218)

**Supplemental Figure 1. Density plots**

**Dotted line indicates data split point**


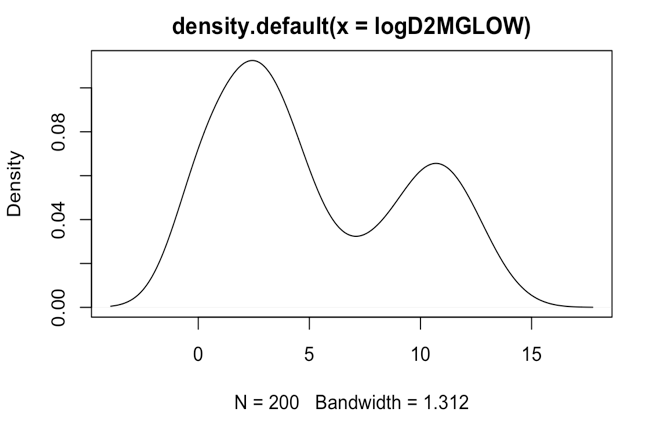
DENV-1 MG LOW DENV-2 MG LOW


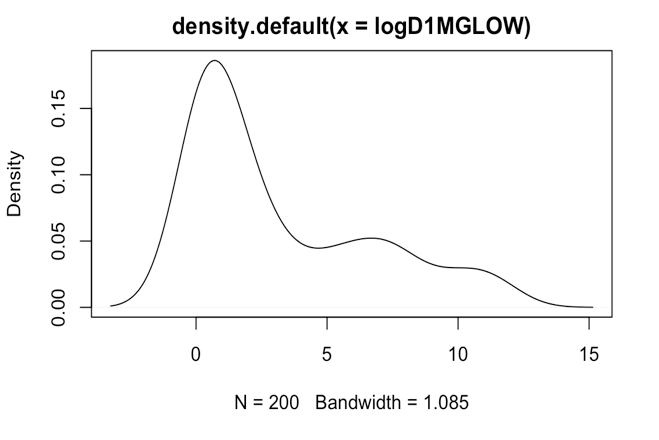


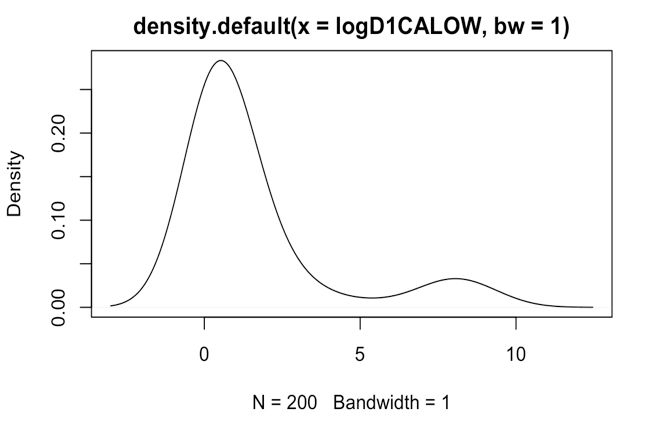
DENV-1 CA LOW DENV-2 CA LOW


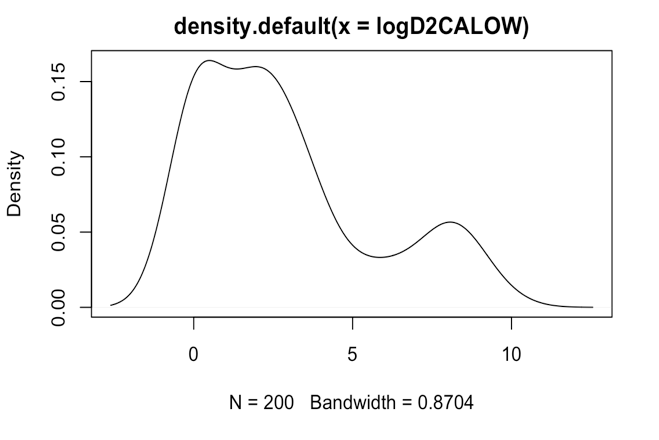


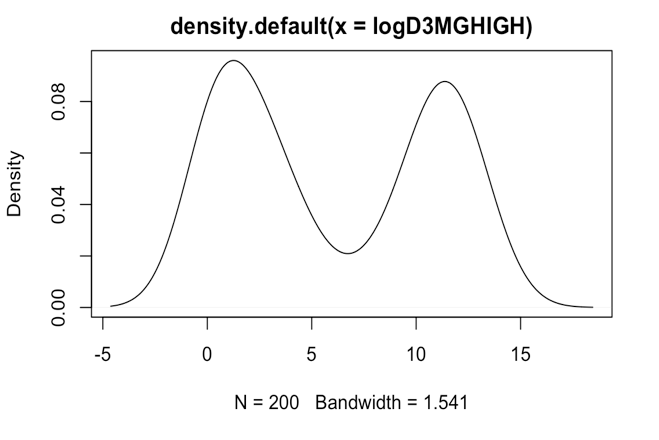
DENV-3 MG HIGH DENV-4 MG HIGH


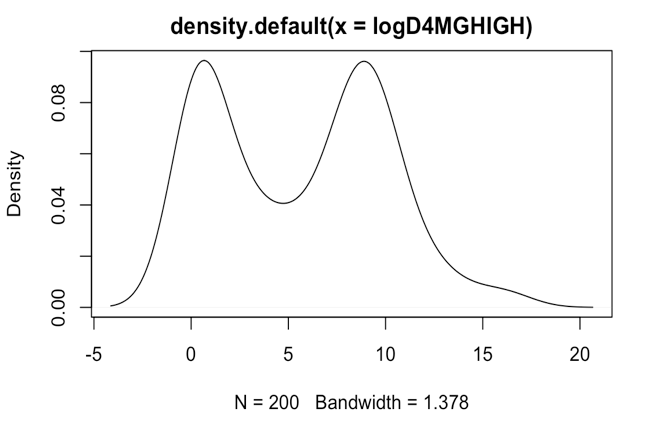


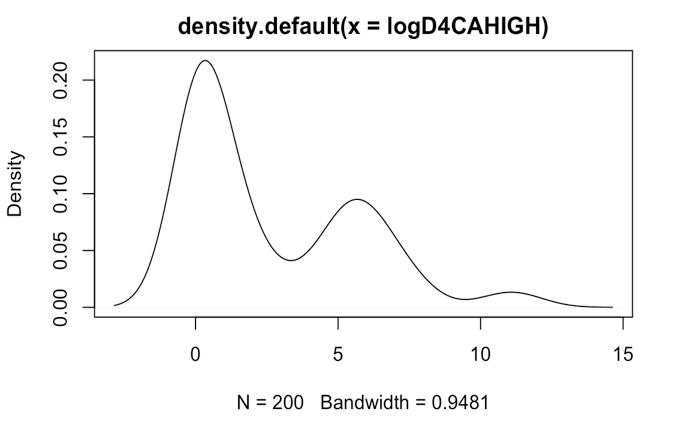

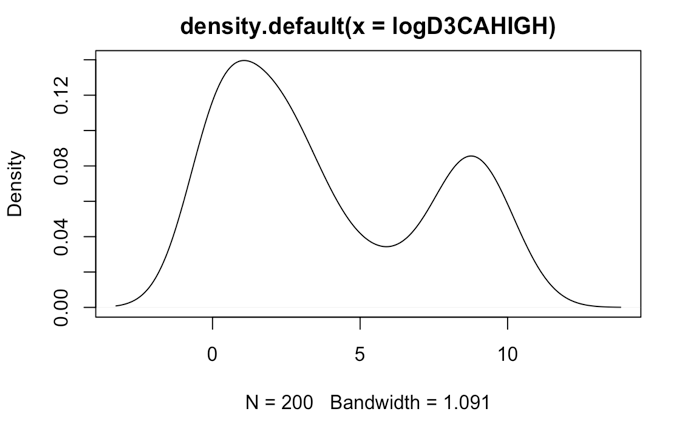
DENV-3 CA HIGH DENV-4 CA HIGH

DENV-1 CA HIGH DENV-2 CA HIGH


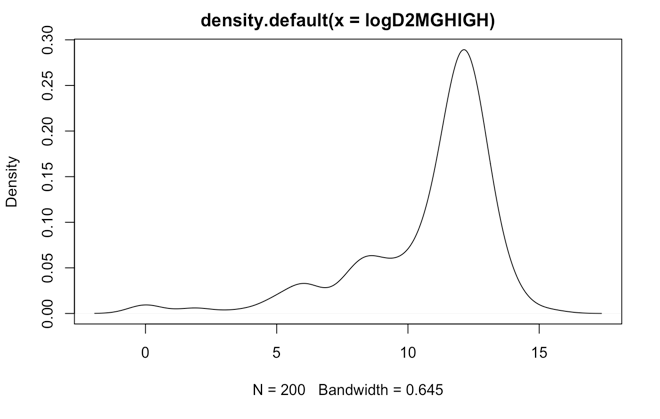

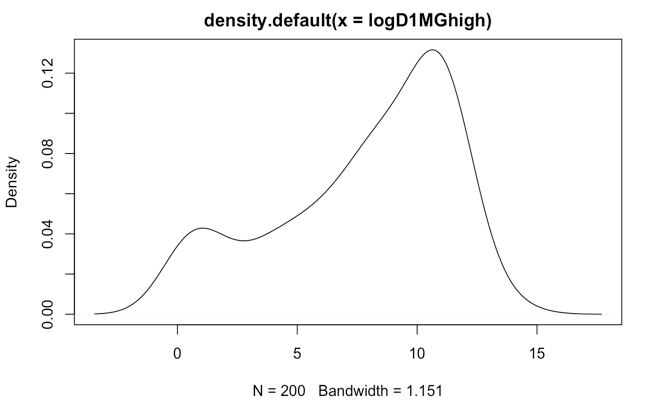


DENV-1 MG HIGH DENV-2 MG HIGH


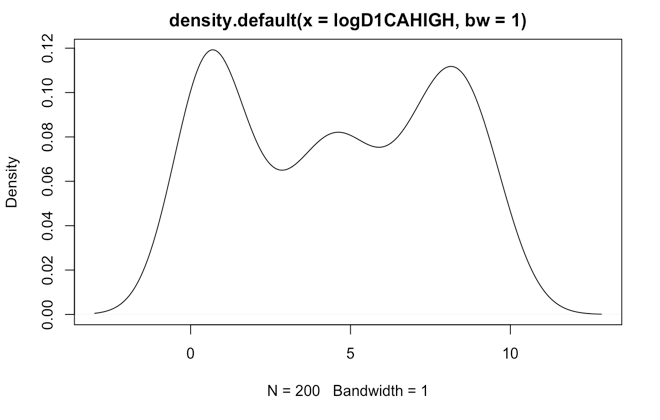

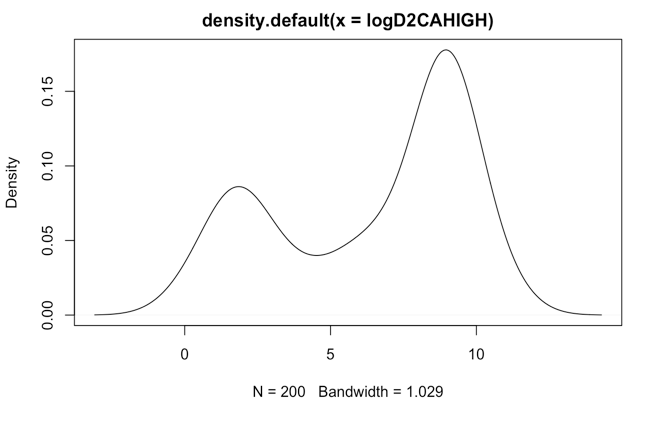

Supplement: S1 Fig — Dotted line indicates data split point. (DOCX) [file ppat.1008218.s006.docx]
