# Supplementary material for: Intra-host growth kinetics of dengue virus in the mosquito Aedes aegypti
Source: PLoS Pathog. 2019 Dec 2;15(12):e1008218. doi: 10.1371/journal.ppat.1008218 (PMC6907869; doi:10.1371/journal.ppat.1008218)
Supplement: S2 Fig — Average DENV load at DPI 20 for all 4 DENV serotypes at 2 infectious doses (High: 1 × 10^8^, low: 1 × 10^5^ DENV copies/ml). All box plots show median and interquartile ranges (n = 10 per treatment). Significant differences are based on Tukey post hoc comparison following ANOVAs on log-transformed data. Only significant differences are shown. *p<0.05. (DOCX) [file ppat.1008218.s007.docx]

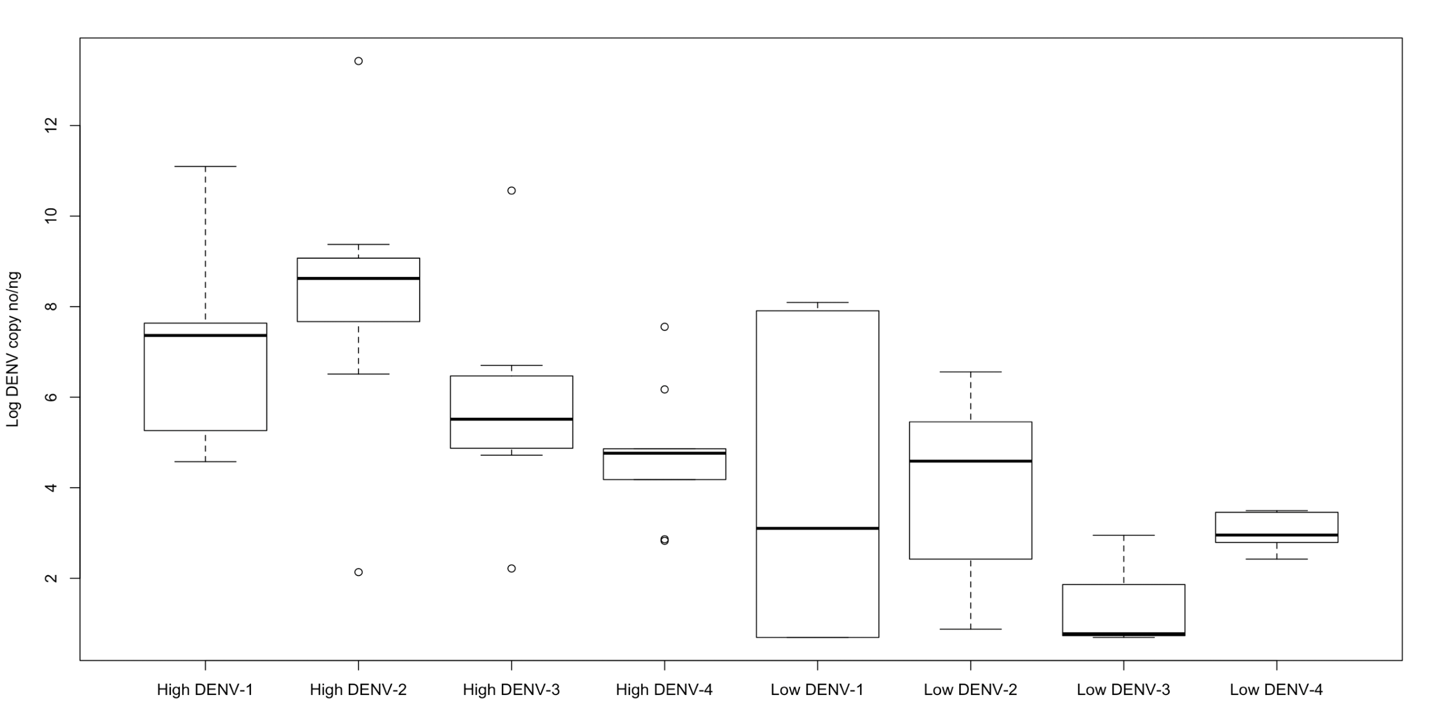
**Supplemental Figure 2. Average salivary gland DENV load at the last DPI (20 days)**

**

Average DENV load at DPI 20 for all 4 DENV serotypes at 2 infectious doses (High: 1 × 10^8^, low: 1 × 10^5^ DENV copies/ml). All box plots show median and interquartile ranges (n = 10 per treatment). Significant differences are based on Tukey *post hoc* comparison following ANOVAs on log-transformed data. Only significant differences are shown. *p<0.05.
