# Supplementary material for: Intra-host growth kinetics of dengue virus in the mosquito Aedes aegypti
Source: PLoS Pathog. 2019 Dec 2;15(12):e1008218. doi: 10.1371/journal.ppat.1008218 (PMC6907869; doi:10.1371/journal.ppat.1008218)
Supplement: S3 Fig — Survival curve analysis of control and the 4 DENV serotypes at 2 infectious doses (High: 1 × 10^8^, low: 1 × 10^5^ DENV copies/ml). There was no significant difference between treatments (χ2 = 12.9, df = 8, p = 0.1). Dotted outside lines represent 95% confidence intervals. Each line at the top represents individual treatments. (DOCX) [file ppat.1008218.s008.docx]

**Supplemental Figure 3. Survival curves for all treatments**


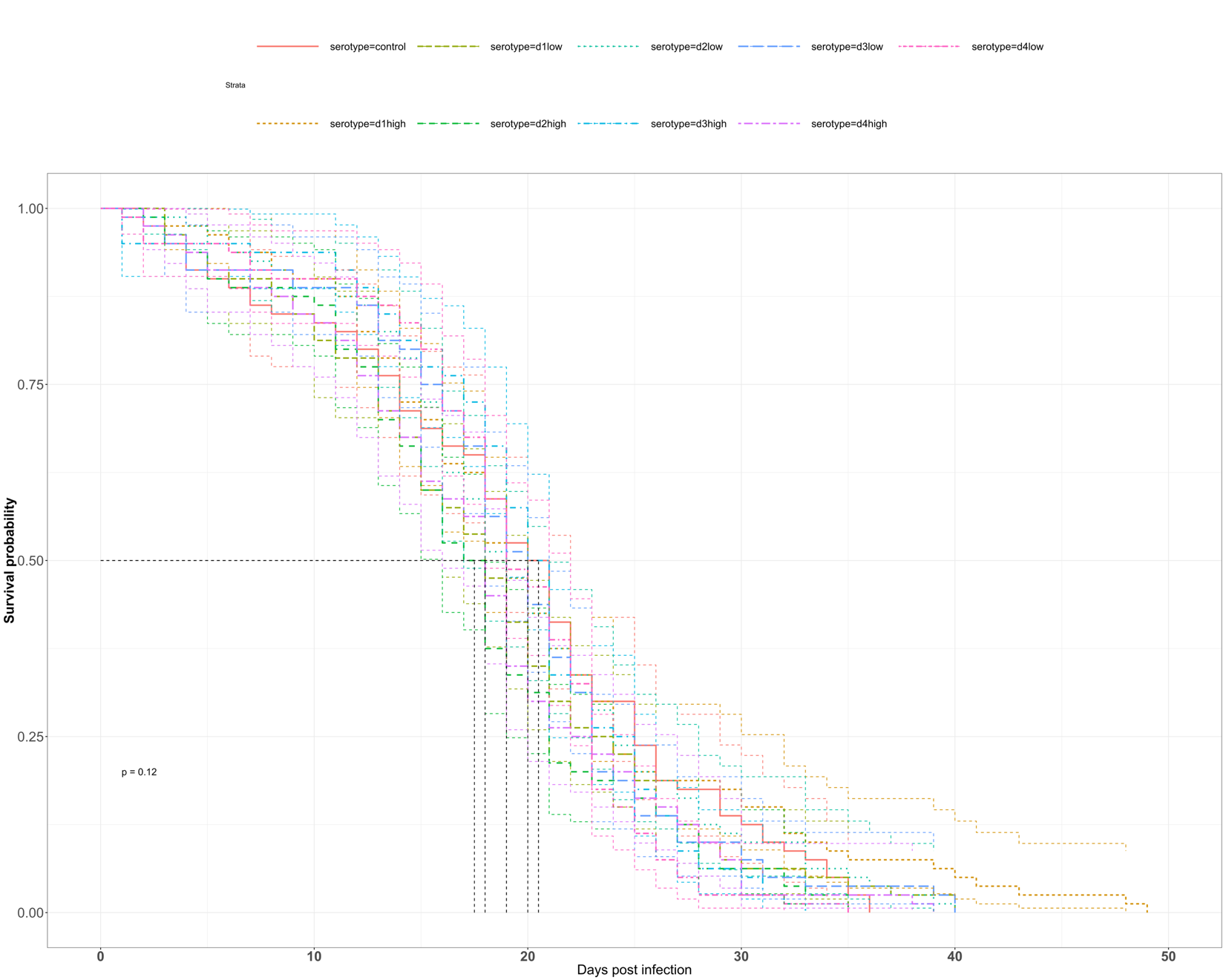


Control

DENV-1

LOW

DENV-2

LOW

DENV-3

LOW

DENV-4

LOW

DENV-1

HIGH

DENV-2

HIGH

DENV-3

HIGH

DENV-4

HIGH

Treatments

Survival curve analysis of control and the 4 DENV serotypes at 2 infectious doses (High: 1 × 10^8^, low: 1 × 10^5^ DENV copies/ml). There was no significant difference between treatments (χ^2^ = 12.9, df = 8, p = 0.1). Dotted outside lines represent 95% confidence intervals. Each line at the top represents individual treatments.
